# Supplementary material for: A Global Screen for Assembly State Changes of the Mitotic Proteome by SEC-SWATH-MS
Source: Cell Syst. 2020 Feb 26;10(2):133–155.e6. doi: 10.1016/j.cels.2020.01.001 (PMC7042714; doi:10.1016/j.cels.2020.01.001)

Q96KP4 | CNDP2_HUMAN | CNDP2 CN2 CPGL HEL−S−13 PEPA
Monomer MW [kDa]: 52.878 Monomer expected elution fraction: 46

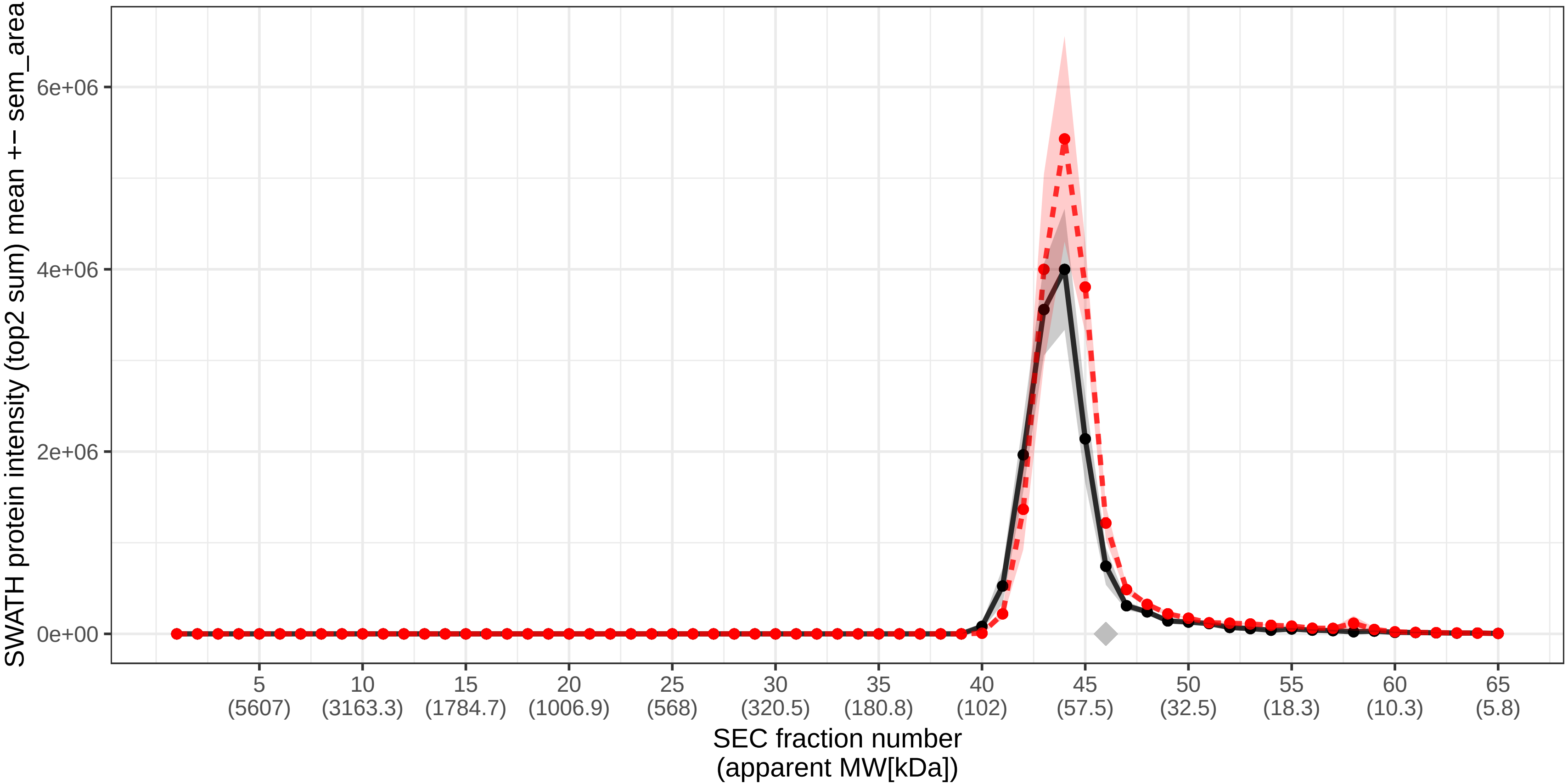

Supplement: Data S1. SEC-SWATH-MS Protein Chromatograms, Related to Figure 1 [file mmc6.zip › SECchrom_Q96KP4_CNDP2_HUMAN_CNDP2_CN2_CPGL_HEL-S-13_PEPA.pdf]
